# Vitamin D supplementation in systemic lupus erythematosus: relationship to disease activity, fatigue and the interferon signature gene expression

**DOI:** 10.1186/s41927-021-00223-1

**Published:** 2021-12-03

**Authors:** Rosalie Magro, Christian Saliba, Liberato Camilleri, Christian Scerri, Andrew A. Borg

**Affiliations:** 1 Rheumatology Department, Mater Dei Hospital, Tal-Qroqq, Msida, MSD 2090 Malta; 2 Faculty of Medicine and Surgery, University of Malta, Msida, Malta; 3 Centre for Molecular Medicine and Biobanking, University of Malta, Msida, Malta; 4 Statistics and Operations Research, Faculty of Science, University of Malta, Msida, Malta

**Keywords:** Vitamin D, Systemic lupus erythematosus, Fatigue, Interferon, Disease activity

## Abstract

**Background:**

In addition to the well-known role of vitamin D in calcium homeostasis and bone metabolism, vitamin D is important in the modulation of the immune system and inflammatory processes. Vitamin D deficiency is common in patients with systemic lupus erythematosus (SLE), possibly as a result of sun avoidance. The aim of this prospective open-label study was to assess the effect of the treatment of vitamin D deficiency and insufficiency in SLE patients, particularly with regards to disease activity, fatigue and interferon signature gene expression.

**Methods:**

31 SLE patients, 13 with vitamin D deficiency and 18 with vitamin D insufficiency were treated with vitamin D3. They were supplemented with vitamin D3 8000 IU daily for 8 weeks if they were vitamin D deficient, or 8000 IU daily for 4 weeks if they were insufficient. This was followed by 2000 IU daily maintenance. They were assessed at baseline, after 6 and 12 months by means of an interview, filling in questionnaires and blood tests. The expression of 12 interferon signature genes in RNA extracted from whole blood was measured by using QuantiGene Plex technology.

**Results:**

An improvement in disease activity measured by systemic lupus erythematosus disease activity index-2K (SLEDAI-2K; *p* = 0.028) and fatigue measured by fatigue severity scale (FSS; *p* = 0.071) at 12 months were noted. A significant decrease in anti-double stranded deoxyribonucleic acid (dsDNA) titre (*p* = 0.045) was also noted. The mean interferon signature gene expression score decreased from baseline to 6 months, however statistical significance was not achieved (*p* = 0.165).

**Conclusions:**

Improved disease activity and fatigue have been noted when Vitamin D has been supplemented in vitamin D deficient/insufficient SLE patients. One possible mechanism could be the suppression of the interferon signature gene expression.

*Trial registration*: The study was registered with the ISRCTN registry on 12/04/2021 (Trial ID: ISRCTN59058825).

## Introduction

Vitamin D is a fat-soluble vitamin present in few foods, mainly in seafood and eggs. The main source (80–90%) of vitamin D is its synthesis in the skin upon absorption of ultraviolet B radiation by 7-dehydrocholesterol. Hydroxylation then occurs in the liver to produce 25-hydroxyvitamin D; this is then hydroxylated further to 1,25-dihydroxyvitamin D. Vitamin D promotes calcium absorption in the gastrointestinal tract and is important to maintain adequate serum calcium and phosphate concentrations. It is also important for bone growth and remodelling [1]. Vitamin D acts through a nuclear receptor [vitamin D receptor (VDR)] that is present in most cells, and it can regulate the transcription of over 200 genes. It thus has multiple additional roles, including neuromuscular and immune functions, modulation of cell growth and reduction of inflammation [2].

Vitamin D deficiency is common in the general population. Studies have shown that 34–67% of adults in Europe are vitamin D deficient (serum 25-hydroxyvitamin D concentrations ≤ 20 ng/mL) [3]. The prevalence of vitamin D deficiency is higher in patients with Systemic Lupus Erythematosus (SLE) patients, most likely due to sun avoidance [4]. An interest on the role of vitamin D in the pathogenesis of SLE has been created with the discovery of the presence of VDR in cells belonging to the innate and adaptive immune systems (including dendritic cells, macrophages, T cells and B cells) [5]. The influence of vitamin D on the course and prognosis of SLE is still unknown.

A significant inverse association between vitamin D and disease activity measurements (including systemic lupus erythematosus disease activity index (SLEDAI), high anti-dsDNA titres and low complement levels) was noted in a systematic review [5]. This was also confirmed in a meta-analysis [6]. Six studies have been identified that have looked at the relationship between vitamin D and fatigue in SLE; three cross-sectional studies, one prospective study and two randomised controlled studies [7–12]. The cross-sectional studies failed to demonstrate a relationship between the two factors, but the prospective study by Ruiz-Irastorza et al. showed a significant improvement in fatigue with vitamin D supplementation. This was confirmed in the two randomised controlled studies.

The aim of this study was to establish any potential benefit, on the level of fatigue, disease activity, sleep quality, functional disability and steroid use, from vitamin D supplementation to SLE patients with vitamin D deficiency or insufficiency. A further aim was to establish whether such potential benefit could be mediated by the effect of vitamin D supplementation on the expression of the interferon (IFN) signature genes. These are genes that are uniquely overexpressed in the peripheral blood of SLE patients, as has been shown by studies on gene expression profiling. This gene expression has a positive correlation with disease activity [13]. A meta-analysis has identified 12 such genes that are either interferon induced or interferon regulated (IFI35, IFIT1, IFIT3, IFITM1, MX1, OAS1, STAT1, STAT2, CCL2, CXCL1, SOCS1, SOCS3) [14].

## Methods

### Patients

SLE patients with vitamin D deficiency (serum 25-hydroxyvitamin D < 20 ng/mL) or insufficiency (serum 25-hydroxyvitamin D 21-29 ng/mL) were identified from the cohort of SLE patients attending Rheumatology clinic at Mater Dei Hospital, Malta. The inclusion criteria included fulfilment of the SLICC classification criteria for SLE, age ≥ 18 years and the presence of vitamin D deficiency/insufficiency [15]. Patients with stage 4 and 5 chronic kidney disease were excluded from the study. Patients who were started on DMARDs or who had an increase in the DMARD dosage during the course of the study were also excluded. 31 SLE patients fulfilled the inclusion and exclusion criteria and gave informed consent to participate in the study; 13 patients had vitamin D deficiency and 18 patients were vitamin D insufficient. The patients were recruited from November 2016 to June 2017. The study was registered with the ISRCTN registry on 12/04/2021 (Trial ID: ISRCTN59058825).

A face-to-face interview conducted by the same physician was carried out with all the participants. The information collected included demographic data, disease duration, drug history, co-morbidities, body mass index, SLE disease activity index (SLEDAI-2K) score and Systemic Lupus International Collaborating Clinics/American College of Rheumatology damage index (SDI). The following questionnaires were filled in by the participants: Fatigue Severity Scale (FSS), Pittsburgh Sleep Quality Index (PSQI), visual analogue scale (VAS) for pain, Hospital Anxiety and Depression Scale (HADS), and modified Health Assessment Questionnaire (mHAQ) [16–19]. These questionnaires were found to be the most appropriate for the purpose of the study [20–23]. The participants could fill in these questionnaires in English or Maltese, depending on their preference [24, 25]. The blood tests carried out included the full blood count, serum 25-hydroxyvitamin D, complement 3 (C3), complement 4 (C4), anti-dsDNA titre, renal function and serum calcium. Urine was tested for urinalysis and microscopy, and for protein creatinine ratio.

Patients with vitamin D insufficiency were advised to take vitamin D3 8000 IU daily for 4 weeks, followed by 2000 IU daily maintenance. Patients with vitamin D deficiency were advised to take vitamin D3 8000 IU daily for 8 weeks, followed by 2000 IU daily maintenance as recommended by guidelines [26].

After 3 months of vitamin D supplementation, serum 25-hydroxyvitamin D level and calcium were checked in order to ensure that adequate vitamin D levels had been achieved and at the same time ensure that the patients did not develop hypercalcaemia. When the desired level of serum 25-hydroxyvitamin D (≥ 30 ng/mL) was not reached, the patients were contacted to check for adherence to the vitamin D supplementation, and when necessary the recommended dosage was increased. The patients were then invited for a repeat assessment after 6 months and after 12 months of vitamin D supplementation. This included repeating the questionnaires, blood and urine tests.

### Measurement of interferon signature gene expression

Two 3 ml blood samples collected in ethylenediaminetetraacetic acid (EDTA) were taken at baseline and after 6 months of vitamin D supplementation in order to measure RNA expression of interferon signature genes in white blood cells. In order to prevent RNA degradation, the blood samples were processed shortly after they were taken. The white blood cells were collected from the blood samples by centrifuging them at 2000×*g* for 15 min at room temperature. RNA was isolated from the white blood cells by using QIAamp® RNA Blood Mini kit according to the manufacturer’s instructions. The concentration and purity of the extracted RNA was measured for each sample by spectrophotometry using a Thermo Scientific NanoDrop™ spectrophotometer. The extracted RNA was stored at − 80 °C at the Biobank at the University of Malta. QuantiGene® Plex Assay kit that uses Luminex® technology, was then used to quantify RNA expression for 12 interferon signature genes (IFI35, IFIT1, IFIT3, IFITM1, MX1, OAS1, STAT1, STAT2, CCL2, CXCL1, SOCS1, SOCS3) and 3 reference genes (RPL 13A, TBP, HPRT1) in the extracted RNA. The median florescence intensity (MFI) results obtained were then normalised according to reference genes, by first subtracting the average background signal for each gene; and then dividing by the geometric mean of the signal obtained for the three housekeeping genes. The IFN signature gene expression score was calculated by first normalising the MFI values for each gene obtained above by subtracting the gene MFI by the minimum MFI for that gene and then dividing by the range of MFI for that gene. The value of the normalised MFI for each gene thus ranged from 0 to 1. The IFN signature gene expression score was calculated by adding the normalised MFI for each of the 12 genes.

### Statistical analysis

All the statistical analysis was carried out using the statistical software IBM SPSS statistics 24. The normality assumption of continuous variables was checked using the Kolmogorov–Smirnov test. For continuous variables that satisfied the normality assumption, the mean and the standard deviation were used to measure central tendency and dispersion; while the median and the range were used for skewed (non-normal) continuous variables. Comparison of continuous variables at baseline and after treatment was carried using the Paired Samples t-test for normally distributed variables and the Wilcoxon signed ranks test for skewed (non-normal) variables. When comparing the mean normalised MFI at baseline and after 6 months for the 12 interferon signature genes, the Bonferroni correction was used to compensate for the multiple testing. Comparison of the characteristics of patients who had a decrease in IFN signature score with those in whom it increased was carried out by using the Independent Samples t-test for normally distributed variables and the Mann Whitney test for non-normally distributed variables.

## Results

### Demographic data

90.3% of SLE patients studied were female and the mean age was 47.9 years (range 22–75 years). The mean serum 25-hydroxyvitamin D level at baseline was 21.7 ng/ml. Out of the 31 patients, 11 patients were already receiving both calcium and vitamin D supplementation at baseline at a mean daily dose of 764 mg and 527 IU respectively. The reasons for this were osteopaenia/osteoporosis in 4 patients, current steroid therapy in 4 patients and both of these reasons in 3 patients. The baseline clinical characteristics of the cohort are summarised in Table 1. Out of the 7 patients on prednisolone (at a mean dose of 6.2 mg daily), 3 patients remained on the same dose throughout the study; 2 patients had a higher dose than baseline in their 6 month assessment but had stopped prednisolone by 12 months; 2 patients had their dose decreased at both assessments compared to baseline.

**Table 1:** Clinical characteristics of the cohort at baseline

Characteristics	Values
Age, mean (S.D.) years	47.9 (13.7)
Female sex, n/N (%)	28/31 (90.3)
Caucasian race, n/N (%)	30/31 (96.8)
Disease duration, mean (S.D.) years	14.1 (8.0)
Age of SLE onset, mean (S.D.) years	33.8 (13.5)
BMI, mean (S.D.) kg/m^2^	29.8 (8.1)
Osteopaenia/osteoporosis, n/N (%)	8/31 (25.8)
Anti-phospholipid syndrome, n/N (%)	3/31 (9.7)
Current prednisolone, n/N (%)	7/31 (22.6)
Current hydroxychloroquine, n/N (%)	18/31 (58.1)
Current azathioprine, n/N (%)	5/31 (16.1)
Current methotrexate, n/N (%)	4/31 (12.9)
Current calcium supplementation, n/N (%)	11/31 (35.5)
Current vitamin D supplementation, n/N (%)	11/33 (35.5)
SLEDAI-2K, median (range)	4 (0–11)
SDI, median (range)	0 (0–3)

Frequencies and percentages describe categorical variables, while measures of central tendency and dispersion describe continuous variables. The mean and standard deviation are used for normally distributed variables, while the median and range are used for non-normally distributed variables

### Patient characteristics at 6 and 12 months

After 6 months of vitamin D supplementation, 83.9% had a sufficient level of vitamin D (≥ 30 ng/mL), 9.7% were insufficient (20–29 ng/mL) and 6.5% were deficient (< 20 ng/mL). At the 12 month assessment, 35.5% had a sufficient level of serum 25-hydroxyvitamin D, 54.8% were insufficient and 9.7% were deficient. Adherence by the patients to vitamin D3 supplements was assessed by counting the number of vitamin D boxes picked up by the patients. 64.5% of participants did not pick up all the tablets required to follow the recommended regime.

The characteristics of the patients after 6 and 12 months of vitamin D3 supplementation were compared to those at baseline (Table 2). An improvement in disease activity as measured by SLEDAI-2K and clinical SLEDAI-2K was noted. This was statistically significant at 12 months of supplementation (*p* = 0.028, *p* = 0.024 respectively). This is in keeping with the observed significant improvement in anti-dsDNA level at both 6 and 12 months (*p* = 0.032, *p* = 0.045). The daily dose of prednisolone and fatigue measured by FSS decreased after 1 year of vitamin D supplementation (*p* = 0.068, *p* = 0.071 respectively), although statistical significance was not reached. On analysing the results of the 13 patients who were vitamin D deficient at baseline, the improvement in SLEDAI-2K was statistically significant not only at 12 months (*p* = 0.046), but also at 6 months (*p* = 0.030).

**Table 2:** Results obtained when the continuous variables at 6 and 12 months are compared with those at baseline

Variable	Time	Mean	SD	*p *value
SLEDAI-2K	Baseline	3.55	2.998	
	6 months	3.06	2.707	0.324*
	12 months	2.45	2.158	0.028*
Clinical SLEDAI-2K	Baseline	1.97	2.614	
	6 months	1.39	2.076	0.258*
	12 months	0.90	1.620	0.024*
FSS	Baseline	4.10	1.514	
	6 months	3.95	1.623	0.465
	12 months	3.70	1.852	0.071
VAS pain	Baseline	3.74	2.966	
	6 months	3.97	3.027	0.603
	12 months	3.35	3.115	0.276
HADS-D	Baseline	4.81	3.674	
	6 months	5.55	3.767	0.084
	12 months	4.48	4.186	0.389
HADS-A	Baseline	8.58	4.703	
	6 months	8.26	4.389	0.499
	12 months	8.29	4.776	0.605
PSQI	Baseline	6.66	4.218	
	6 months	6.52	4.396	0.943
	12 months	6.16	4.352	0.289
mHAQ	Baseline	0.23	0.357	
	6 months	0.29	0.402	0.146*
	12 months	0.28	0.444	0.111*
Corrected calcium (2.05–2.60 mmol/l)	Baseline	2.30	0.092	
	6 months	2.28	0.085	0.139
	12 months	2.29	0.092	0.647
25-hydroxyvitamin D (30–100 ng/mL)	Baseline	21.74	6.444	
	6 months	32.61	6.396	**< 0.001**
	12 months	28.32	8.368	**0.001**
Anti-dsDNA level (0.0–100 IU/mL)	Baseline	172.26	184.217	
	6 months	154.99	151.173	0.032*
	12 months	154.11	153.276	0.045*
Prednisolone daily dose (mg)	Baseline	1.403	2.850	
	6 months	1.550	2.973	0.144*
	12 months	0.937	2.459	0.068*

The *p* values have been obtained using the Paired samples *t* test for normally distributed variables and the Wilcoxon signed ranks test for non-normally distributed variables (as denoted by *). Statistically significant results are highlighted in bold

A sub-group analysis was carried out on the results obtained after 12 months of vitamin D supplementation by including only the 11 patients who achieved the target serum 25-hydroxyvitamin D of ≥ 30 ng/ml. The results showed a statistically significant improvement in FSS (*p* = 0.011) and PSQI (*p* = 0.004) (Table 3).

**Table 3:** Results obtained for 11 patients who obtained target serum 25-hydroxyvitamin D (≥ 30 ng/mL) at 12 months

Variable	Time	Mean	SD	*p* value
SLEDAI-2K	Baseline	4.18	3.545	0.127
	12 months	2.27	1.794	
Clinical SLEDAI-2K	Baseline	2.18	2.892	0.140*
	12 months	0.55	1.293	
FSS	Baseline	4.27	1.261	**0.011**
	12 months	3.33	1.930	
VAS pain	Baseline	3.82	2.639	0.071
	12 months	2.64	2.335	
PSQI	Baseline	6.64	2.908	**0.004**
	12 months	4.73	2.611	
Serum 25-hydroxyvitamin D (ng/ml)	Baseline	23.45	6.138	**< 0.001**
	12 months	37.27	5.293	
Anti-dsDNA level (IU/ml)	Baseline	243.15	200.697	**0.027**
	12 months	198.88	167.210	

The *p* values have been obtained using the Paired samples *t* test for normally distributed variables and the Wilcoxon signed ranks test for non-normally distributed variables (as denoted by *). Statistically significant results are highlighted in bold

### Interferon signature gene expression

The mean normalised MFI for the 12 interferon signature genes at baseline was compared with that after 6 months of vitamin D3 supplementation. Since multiple genes were tested, a significance level for the p value of 0.004 was established using the Bonferroni correction. The mean normalised MFI decreased from baseline to 6 months of vitamin D3 supplementation for the expression of all 12 interferon signature genes especially SOCS1 and OAS1 genes (*p* = 0.008 and 0.039 respectively).

The mean IFN signature gene expression score decreased from 2.666 (SD 1.703) at baseline to 2.225 (SD 1.323) after 6 months of vitamin D supplementation (*p* = 0.165). The characteristics of the patients in whom a decrease in IFN signature gene expression score was noted (17 patients) were compared with those who did not have a decrease in score (12 patients) from baseline to 6 months. The patients who had a decrease in their IFN signature gene expression score had a significant lower SLEDAI-2K at 6 months (mean SLEDAI-2K 2.06) than those who had an increase in IFN signature gene expression score (mean SLEDAI-2K 4.67; *p* = 0.015).

## Discussion

This prospective open-label study has followed up 31 SLE patients with vitamin D deficiency/insufficiency for 1 year. It has shown that with vitamin D3 supplementation, there has been an improvement in disease activity measured by SLEDAI-2K. This was statistically significant after 12 months of vitamin D supplementation (*p* = 0.028). In keeping with this the serum anti-dsDNA level decreased at both 6 months (*p* = 0.032) and at 12 months (*p* = 0.045) when compared to baseline. Moreover there was a decrease in the prednisolone dose and level of fatigue at 12 months. However these did not achieve statistical significance (*p* = 0.068, *p* = 0.071), most likely due to the limited sample size. The decreased adherence to vitamin D supplementation could have masked the improvement in fatigue (*p* = 0.011) and sleep quality (*p* = 0.004) which were apparent on analysis of a sub-group of patients who achieved normal level of serum 25-hydroxyvitamin D at 12 months. No changes in the level of depression, anxiety and functional disability were noted. Despite having a higher percentage of patients who achieved target serum 25-hydroxyvitamin D level at 6 months when compared to 12 months, the improvement in disease activity and fatigue was more evident at 12 months. This suggests that a period of vitamin D supplementation longer than 6 months is necessary in order to achieve the maximal benefits on disease activity and fatigue.

Patients included in the study did not have any increase in DMARD dosage and were not started on any new DMARDs or biological agents during the course of the study to ensure that the observed improvement was not due to the effect of these drugs. Two patients had a slight increase in prednisolone dosage from baseline to 6 months by 1.25 mg and 1.5 mg respectively, however in both cases prednisolone was stopped before the 12 month assessment. Hence the observed improvement from baseline to 12 months was not due to the effect of prednisolone.

This study made use of the QuantiGene Plex technology, which enabled the simultaneous assessment of the expression of multiple genes from RNA extracted from white blood cells in SLE patients at baseline and after 6 months of vitamin D3 supplementation. The results showed that the mean MFI of all 12 interferon signature genes assessed, decreased after vitamin D3 supplementation. This was not deemed as statistically significant in view of the Bonferroni correction that was applied. The IFN signature gene expression score decreased from baseline to 6 months, however statistical significance was not achieved likely due to the limited sample size (*p* = 0.165). On comparing the characteristics of the patients in whom a decrease in IFN signature gene expression score was noted from baseline to 6 months, with those who did not have a decrease in score, no significant differences were noted with the exception of a lower SLEDAI-2K at 6 months in the patients who had a decrease in their score. Future studies should focus on characteristics that may influence the effect by vitamin D on interferon signature gene expression, such as VDR polymorphisms.

Two cross-sectional case control studies showed that in SLE patients, serum 25-hydroxyvitamin D had a significant negative correlation with plasma IFN-α and levels of IFN-α gene expression [27, 28]. Another cross-sectional case control study by Ritterhouse et al. [29] showed that SLE patients with vitamin D deficiency had a significantly higher mean serum IFN-α activity (as measured by the expression of the genes MX1, PKR and IFIT) than those without vitamin D deficiency. A randomized controlled trial did not show a significant effect on the expression of the genes MX1, IFIT1 and IFI44 at 12 weeks following vitamin D supplementation of vitamin D deficient SLE patients at a dose of 2000 IU or 4000 IU daily [30]. This could have been due to the fact that 48.5% of patients supplemented with vitamin D did not obtain the target level of 25-hydroxyvitamin D above 30 ng/ml since a loading dose was not used. In fact, the authors concluded that in order to suppress the expression of the IFN signature genes, higher levels of serum 25-hydroxyvitamin D, maintained for a longer time may be necessary. For this reason interferon signature gene expression was measured after 6 months of vitamin D supplementation in our study.

This study is the first prospective study to assess the change in the expression of all 12 interferon signature genes that are overexpressed in SLE, as identified by meta-analysis, with vitamin D treatment [14]. The use of a vitamin D3 loading dose and the measurement of serum 25-hydroxyvitamin D after 3 months of supplementation, enabled a higher proportion of patients to achieve the target 25-hydroxyvitamin D of ≥ 30 ng/mL. This allowed the changes in disease activity, interferon signature gene expression and other measured modalities to become more evident after 6 months of treatment.

Vitamin D3 supplementation at the dosage used in this study was noted to be safe. No cases of hypercalcaemia were observed. Adherence to the vitamin D supplements was assessed according to the amount of supplements collected by the patients. These were provided to the patients for free, to encourage adherence. Despite this, a high proportion of patients did not collect all the tablets, according to the recommended regime. Even though the patients could have obtained the supplementation from elsewhere, if this was more convenient for them, the results suggest that a significant amount of patients were not fully adherent particularly towards the end of the 1 year period studied. This is also suggested by the fact that even though the target serum 25-hydroxyvitamin D level of ≥ 30 ng/mL was achieved by 83.9% of patients at 6 months, this dropped to 35.5% at 12 months.

The measurement of fatigue is not straightforward and a number of instruments have been used to determine the level of fatigue [31]. The Ad Hoc Committee on Systemic Lupus Erythematosus Response Criteria for Fatigue was set up to establish the instrument of choice when measuring fatigue in SLE [20]. Fifteen such instruments were reviewed and the instrument that was recommended was the Fatigue Severity Scale (FSS) [16]. This questionnaire was originally developed for use in SLE and multiple sclerosis, but has also been used in other medical conditions. The FSS is an easy, self-reported questionnaire consisting of nine statements related to fatigue. Each statement is scored from one to seven; the higher the agreement to the statement the higher the score. The mean of the nine statements is calculated to make up the final FSS score. This instrument of choice in the assessment of fatigue in SLE, was used in this study. The aetiology of fatigue is multifactorial and includes behavioural factors (such as physical activity), co-morbidities (including pain, depression, anxiety and sleep disorder) and medication side effects (including anti-depressants) [32–34]. Changes in factors influencing fatigue, such as lifestyle changes and change in anti-depressant medication, could have impacted the measured fatigue.

The summer months were avoided on recruiting patients in order to minimise the potential seasonal effect of (1) higher temperatures on increasing fatigue and (2) higher sun exposure on baseline vitamin D levels. By using a 12 month period of vitamin D supplementation, the potential effect of seasonality on fatigue was reduced. During the course of the study serum 25-hydroxyvitamin D was used as the measure of vitamin D eliminating the effect of sunlight as a potential confounding factor. 1,25-dihydroxyvitamin D is the active form of vitamin D, which binds to the vitamin D receptor and propagates its effect on the cells of the immune system. This active form is not routinely measured since its serum levels tend to fluctuate and 25-hydroxyvitamin D has been used as the measure of serum vitamin D. This complicates the interpretation of the effect of vitamin D, particularly since the hydroxylation of 25-hydroxyvitamin D to 1,25-hydroxyvitamin D is influenced by factors such as drugs (e.g. hydroxychloroquine) and renal disease [35]. A further limitation of this study, as with any open-label study, is the lack of a placebo controlled group. This could have introduced an element of bias in the assessments by the assessor or the participants, particularly in the filling of the self-assessment questionnaires. This is less so however, in the laboratory results including the measurement of gene expression. Larger studies including well designed, randomised double blind placebo controlled trials on the effect of vitamin D supplementation in SLE, would provide a stronger level of evidence.

## Conclusions

In conclusion, this study supports the screening of SLE patients for vitamin D deficiency. This is common due to sun avoidance and use of sunscreen, as well as due to renal insufficiency in patients with lupus nephritis. The correction of vitamin D deficiency in SLE patients has potential benefits with regards to the suppression in the expression of genes in the interferon pathway, consequentially resulting in an improvement in SLE disease activity. Other potential benefits include the improvement of sleep quality and fatigue; although the factors related to fatigue in SLE are complex, and further studies in this respect are required.

## Data Availability

The datasets used and/or analysed during the current study are available from the corresponding author on reasonable request.

## Abbreviations

C3: Complement 3
C4: Complement 4
CCL2: C–C Motif Chemokine Ligand 2
CXCL1: C-X-C Motif Chemokine Ligand 1
DMARD: Disease modifying anti-rheumatic drugs
dsDNA: Double stranded deoxyribonucleic acid
EDTA: Ethylenediaminetetraacetic acid
FSS: Fatigue Severity Scale
HADS: Hospital Anxiety and Depression Scale
HPRT1: Hypoxanthine Phosphoribosyltransferase 1
IFI35: Interferon Induced Protein 35
IFIT1: Interferon Induced Protein with Tetratricopeptide Repeats 1
IFIT3: Interferon Induced Protein with Tetratricopeptide Repeats 3
IFITM1: Interferon Induced Transmembrane Protein 1
IFN: Interferon
MFI: Median florescence intensity
mHAQ: Modified Health Assessment Questionnaire
MX1: MX Dynamin like GTPase 1
OAS1: 2′–5′-Oligoadenylate Synthetase 1
PSQI: Pittsburgh Sleep Quality Index
RNA: Ribonucleic acid
RPL 13A: Ribosomal Protein L13a
SDI: Systemic Lupus International Collaborating Clinics/American College of Rheumatology damage index
SLE: Systemic lupus erythematosus
SLEDAI: Systemic lupus erythematosus disease activity index
SOCS1: Suppressor of Cytokine Signaling 1
SOCS3: Suppressor of Cytokine Signaling 3
STAT1: Signal transducer and activator of transcription 1
STAT2: Signal transducer and activator of transcription 1
TBP: TATA-Box Binding Protein
VAS: Visual analogue scale
